# Hemodialysis Fatigue: Just “Simple” Fatigue or a Syndrome on Its Own Right?

**DOI:** 10.3389/fphys.2012.00306

**Published:** 2012-07-31

**Authors:** Giorgos K. Sakkas, Christina Karatzaferi

**Affiliations:** ^1^Center for Research and Technology ThessalyTrikala, Greece; ^2^Department of Physical Education and Sports Science, University of ThessalyTrikala, Greece

## Introduction

In this opinion article we discuss whether fatigue symptoms and signs often noticed in the hemodialysis (HD) patients should be collectively viewed and addressed as a “syndrome” and not in usual polarities such as peripheral vs. central fatigue, or, physical vs. mental fatigue etc. Our rationale follows the example of “Chronic Fatigue Syndrome (CFS)” to help us substantiate our argument. It is important to stress that “Sign” and “Symptom” are terms with different meanings; symptoms are problems that a patient perceives while signs are whatever features an observer can objectively detect or measure. In practice a syndrome combines symptoms, signs, and phenomena that the health practitioner recognizes, being alerted in that if some are observed then more are expected. In the case of what is being increasingly described as “HD fatigue” symptoms and signs may not have a very clear association resulting in ineffective or no action to remedy, partly because we have not yet identified/labeled them, as a scientific or health care community, as a group, i.e., as a syndrome.

Chronic renal disease is a “silent epidemic” affecting up to 10% of the population in the USA, and Asian countries, with some sufferers progressing in to end-stage renal failure (Stenvinkel, 2010). Renal disease patients are characterized by progressively worsening muscle weakness and muscle atrophy due to both a metabolic and a disuse component collectively described as uremic myopathy. While various interventions in stable HD patients have helped these patients improve their functionality, they still have not proven enough to bring their muscle quality and quantity up to the levels of a healthy sedentary person (Sakkas et al., 2003b; Johansen et al., 2006). Moreover, patients present with sleep problems (Sakkas et al., 2008a), neurological and quality of life issues (Giannaki et al., 2011), anxiety, and/or symptoms of depression but most notably they complain of chronic fatigue and “lack of energy” (McCann and Boore, 2000).

## What is Fatigue?

The traditional approach in identifying, researching, and assessing signs of fatigue has a dual nature: central vs. peripheral, brain vs. muscle, physical vs. mental etc.

Mental fatigue is a psychobiological state caused by prolonged and intensive cognitive activity and is expressed by the lack of concentration and the inability of staying focused under certain conditions (Marcora et al., 2009). Physical (muscle) fatigue on the other hand is accepted mainly as an inability to exert or sustain muscle force or power output for a given task (Edwards, 1975). Moreover, there is heated discussion in the literature on the origins and modulation of physical fatigue (Shephard, 2009; Noakes, 2011a,b), beyond the scope of this article.

Likewise in chronic disease patients, it is been suggested that the symptoms of fatigue relate to two components: the mental that encompasses emotional and cognitive qualities and the physical, encompassing sleepiness, lack of energy, and muscle weakness (Hardy and Studenski, 2010).

How we view fatigue is of course important as it dictates how we identify signs, how we interpret or link symptoms and how we implement rehabilitation interventions and overall patient care. We can conclude that “*fatigue is the inability of sustaining an effort either mentally or physically or both while signs and symptoms may be interconnected in a way not always clearly defined*.”

## Fatigue in Hemodialysis Patients

While HD *per se* is a life saving procedure, it cannot substitute for a healthy kidney, it taxes the patient and HD related fatigue symptoms significantly affect patients’ quality and way of life as suggested by many (McCann and Boore, 2000; Caplin et al., 2011; Gordon et al., 2011; Jhamb et al., 2011). Muscle status is gravely affected with HD patients demonstrating severe atrophy, fat infiltration, and other anomalies (Sakkas et al., 2008b). Overall, HD patients exhibit low levels of physical activity and functional capacity while they suffer from generalized weakness, exercise intolerance, and muscle atrophy, all leading to generalized sense of fatigue (Johansen and Painter, 2012). The causes of fatigue in HD patients are not well understood but it is been shown that these should include both muscular and central activation failures (Johansen et al., 2005).

Moreover, correction of anemia (Jhamb et al., 2011), exercise training (Koufaki et al., 2002; Sakkas et al., 2003b) with or without anabolic steroid (Johansen et al., 2006), and/or nutritional supplements (Kalantar-Zadeh et al., 2011) do improve the clinical condition of the HD patients, however, despite such improvements, we have observed that HD patients’ functional capacity cannot equal that of a sedentary aged matched healthy counterpart.

A cause of the observed minimal levels of physical activity is probably that the HD procedure *per se* [e.g., duration of dialysis sessions (Caplin et al., 2011) etc.,] contributes to fatigue. One third of the patients report that they feel worse in the immediate hours after the dialysis session while one out of four reports severe or very severe intensity of fatigue after dialysis (Gordon et al., 2011). The severity of “Post-dialysis Fatigue” symptoms could range from mild to severe and can last from a few hours after the dialysis procedure up to until the next day (Lindsay et al., 2006) or for a “very long time” (Gordon et al., 2011). Thus many HD patients may spent a large proportion of their time in a state of fatigue (Caplin et al., 2011; Gordon et al., 2011), and since they perceive fatigue (whether in dialysis or in non-dialysis days) as an important barrier (Delgado and Johansen, 2012), this adversely affects their physical activity levels.

Other factors that contribute to the excessive fatigue area lack of restorative sleep (Sakkas et al., 2008b), excess pre-dialysis weight (Sklar et al., 1999), poor nutritional status (Jhamb et al., 2011), restless legs syndrome (Giannaki et al., 2011), and the overall mental status of the patients (Jhamb et al., 2011). Evidently, of all of these factors can contribute to a self-exacerbating process, a vicious circle, of fatigue due to inactivity and further inactivity due to fatigue. This sensation of an enduring fatigue interferes with physical and social activities and feeds perceptions of increased restrictions and barriers (Delgado and Johansen, 2012), and leads to a significant reduction of physical activity and functional capacity, which in turn contributes to the increased cardiovascular risk and a high mortality rate among these patients (Sarnak et al., 2003).

The state of current knowledge regarding the differences between generalized fatigue and HD treatment related fatigue is not well understood however many variables have been implicated in the severity and the prevalence of the symptoms. The variables can be divided into: (a) social and demographic including age, dialysis vintage, gender, and race, (b) clinical variables including anemia, anorexia, nutritional status, chronic inflammation, sleep disorders, physical inactivity, comorbidities, and depression, and (c) some laboratory variables including Kt/V, serum creatinine and urea, and levels of parathyroid hormone (Bossola et al., 2011). The etiology of fatigue in HD patients is not a simple “one stop” investigation. It involves many aspects of patients’ health as well as various social and behavioral factors that depend on patients’ health characteristics and mental attitude (McCann and Boore, 2000).

## What We Can Learn from Chronic Fatigue

A disorder called “CFS” is generally defined by persistent mental and physical fatigue accompanied by other specific symptoms (Arnett et al., 2011). While its causes are undefined, it is managed as a syndrome (given some accepted “operational” diagnostic criteria; Wessely, 2001) and sufferers are offered multidisciplinary care.

Patients on maintenance HD therapy share many similarities to those suffering by CFS since they experience generalized weakness (Johansen et al., 2003), exercise in tolerance (Koufaki et al., 2002), and disturbed sleep (Sakkas et al., 2008a) all leading to a sense of generalized fatigue and “lack of energy” (McCann and Boore, 2000; Kovacs et al., 2011). This chronic state of “HD Fatigue” among HD patients satisfies one major requirement for the diagnosis of CFS which is persistent fatigue present at least during 50% of the time over a period of at least 6 months (Jason et al., 2003). However, as renal failure is present, the second requirement for the diagnosis of CFS, which is the absence of disease, is contradicted. So far the single symptom approach of fatigue in HD patients did not succeed to ameliorate patients’ sense of fatigue (Letchmi et al., 2011) and therefore, by viewing signs and symptoms of fatigue in a holistic approach would at least allow practitioners and scientists to address the problem as efficiently as in CFS patients. Such an approach will be challenging, given the variety in intensity and the causes of these symptoms, but not impossible, and can hold large benefits to the patients’ quality of life as the CFS treatments have shown so far in other populations.

## Hypothesis of “Hemodialysis Fatigue Syndrome”

The prevalence of general undefined fatigue in HD patients ranges from 30 to 80% depending on the assessment tools and the dialysis modality (Bossola et al., 2011). The average score of fatigue in HD patients is the worst of all chronic disease patients (Ware et al., 1993) including those with severe depression (Yatham et al., 2004), cancer patients undergoing chemotherapy (Adamsen et al., 2009), and lupus patients (Jolly, 2005). In addition, the majority of HD patients complain of various “non-specific” symptoms that are very often considered by their health care providers as “irrelevant” to fatigue. However, if those “irrelevant” symptoms (see below) could be seen under the prism of a “syndrome” (as in the case of CFS), it is possible that the final diagnosis and treatment of symptomatology would be much different.

For example, in the diagnosis of CFS, various complementary criteria play an important role in the final decision such as pain in multiple joints, headaches, nausea, chest pain, shortness of breath, difficulties in maintaining upright position, and various psychological problems such as depression, irritability, mood swings, and other (Burton et al., 2009). Those are evident in HD patients however, the clinical significance of those symptoms changes when viewed in relation to fatigue.

The realization of any physical activity requires an efferent action to recruit the proper number of motor units in the active muscles. At the beginning of the activity, the extent of muscle recruitment will depend mostly on the physical health of the person (Hettinga et al., 2011), the mental and emotional health, the extent of mental fatigue (Marcora et al., 2009), the level of sleep deprivation (Skein et al., 2011), the quality of rest, and recovery from a previous activities (Eston et al., 2007) as well as the level of motivation and self-esteem (Micklewright et al., 2010).

Hypothetically, when the same consideration applies to a HD patient, one should consider that a lower number of motor neurons would be activated due to uremic neuropathy (Krishnan and Kiernan, 2007), that the HD patient may be experiencing muscle atrophy and/or fat infiltration (Sakkas et al., 2003a, 2008b; thus will be weaker than an aged matched control), in addition to various emotional and mental distresses that seem to affect the level of exercise tolerance (Kouidi et al., 2010). Furthermore, the majority of HD patients complain of “brain fog” and lethargy (Caplin et al., 2011) especially in the hours post dialysis (Tryc et al., 2011), while one out of two have very low quality of sleep and suffer from daily sleepiness (Sakkas et al., 2008a; Giannaki et al., 2011). Moreover, the HD procedure *per se* filters away many small molecules involved in metabolism (glucose, amino acids, minerals, hormones etc.) leaving the patient feeling drained and exhausted, with inadequate time to recover, since the next dialysis will take place in less than 48 h (Lim and Flanigan, 1989). Finally, the high hospitalization rate (Collins et al., 2009), the proneness to infection (Kallen et al., 2010), and the feeling of being hospital-bound and stuck to a dialysis machine reduces significantly the level of motivation and the degree of self-esteem (Hedayati et al., 2012). Eventually for an HD patient a simple physical activity, such as a walk around the block assumes a totally different meaning!

## Can a “Hemodialysis Fatigue Syndrome” Exist?

The diagnosis of “CFS” is based on Fukuda's criteria (Fukuda et al., 1994) and includes: unexplained persistent or intermittent chronic fatigue that is of new or definable onset, fatigue not the result of an identified ongoing exertion, not ameliorated by rest and that results in reduced participation in personal, social, or professional activities (Fukuda et al., 1994; Arnett et al., 2011).

According to this definition and diagnosis, a significant proportion of the HD patients may be considered to suffer from a “type of” CFS that remains undiagnosed and untreated. Even though there is not yet scientific evidence suggesting that HD patients’ fatigue symptoms are related to the CFS, anecdotal reports from patients, and clinical practitioners suggest that the sense of fatigue experienced by HD patients is more a syndrome than a single symptom secondary to other causes.

## Perspective and Research Directions

We believe that the current descriptions of HD fatigue point to a “syndrome.” In current practice, treatment for fatigue symptoms and signs, if offered, and dependent on the country and type of care facility, is given mostly by the nephrologists and sometimes by the nursing staff. We believe that to address such complex symptomatology necessitates a more holistic approach which should include other specializations such as neurologists, psychiatrists, exercise physiologists, pain management specialists, and others. It is difficult to effectively treat symptoms such as “lack of energy” and signs such as “muscle weakness” as it is obvious that they cannot be treated fragmentarily, e.g., only with pain killers or antidepressants. A multidisciplinary approach is needed and we propose that while challenging, to formulate, it will be rewarding for patients, health care providers, and scientists.

Future research needs to focus on clinical and functional examination of those HD patients with severe and persistent fatigue vs. those with no or minimal signs and symptoms and clarifying whether or not HD Fatigue may be “equivalent” to CFS. This could lead to better care for HD patients.

## References

[B1] Adamsen L. Quist M. Andersen C. Moller T. Herrstedt J. Kronborg D. Baadsgaard M. T. Vistisen K. Midtgaard J. Christiansen B. Stage M. Kronborg M. T. Rorth M. (2009). Effect of a multimodal high intensity exercise intervention in cancer patients undergoing chemotherapy: randomised controlled trial. BMJ 339, b3410 10.1136/bmj.b3410 19826172 PMC2762035

[B2] Arnett S. V. Alleva L. M. Korossy-Horwood R. Clark I. A. (2011). Chronic fatigue syndrome – a neuroimmunological model. Med. Hypotheses 77, 77–83 10.1016/j.mehy.2011.03.030 21474251

[B3] Bossola M. Vulpio C. Tazza L. (2011). Fatigue in chronic dialysis patients. Semin. Dial. 24, 550–555 10.1111/j.1525-139X.2011.00956.x 21917000

[B4] Burton C. Knoop H. Popovic N. Sharpe M. Bleijenberg G. (2009). Reduced complexity of activity patterns in patients with chronic fatigue syndrome: a case control study. Biopsychosoc. Med. 3, 7 10.1186/1751-0759-3-7 19490619 PMC2697171

[B5] Caplin B. Kumar S. Davenport A. (2011). Patients’ perspective of haemodialysis-associated symptoms. Nephrol. Dial. Transplant. 26, 2656–2663 10.1093/ndt/gfq763 21212166

[B6] Collins A. J. Foley R. N. Gilbertson D. T. Chen S. C. (2009). The state of chronic kidney disease, ESRD, and morbidity and mortality in the first year of dialysis. Clin. J. Am. Soc. Nephrol. 4(Suppl. 1), S5–S11 10.2215/CJN.05980809 19996006

[B7] Delgado C. Johansen K. L. (2012). Barriers to exercise participation among dialysis patients. Nephrol. Dial. Transplant. 27, 1152–1157 10.1093/ndt/gfr404 21795245 PMC3289894

[B8] Edwards R. H. T. (1975). Muscle fatigue. Postgrad. Med. J. 51, 137–143 10.1136/pgmj.51.593.1371219672

[B9] Eston R. Faulkner J. St Clair Gibson A. Noakes T. Parfitt G. (2007). The effect of antecedent fatiguing activity on the relationship between perceived exertion and physiological activity during a constant load exercise task. Psychophysiology 44, 779–786 10.1111/j.1469-8986.2007.00558.x 17617170

[B10] Fukuda K. Straus S. E. Hickie I. Sharpe M. C. Dobbins J. G. Komaroff A. (1994). The chronic fatigue syndrome: a comprehensive approach to its definition and study. International chronic fatigue syndrome study group. Ann. Intern. Med. 121, 953–959 7978722 10.7326/0003-4819-121-12-199412150-00009

[B11] Giannaki C. D. Sakkas G. K. Karatzaferi C. Hadjigeorgiou G. M. Lavdas E. Liakopoulos V. Tsianas N. Koukoulis G. N. Koutedakis Y. Stefanidis I. (2011). Evidence of increased muscle atrophy and impaired quality of life parameters in patients with uremic restless legs syndrome. PLoS ONE 6, e25180 10.1371/journal.pone.0025180 21984901 PMC3184961

[B12] Gordon P. L. Doyle J. W. Johansen K. L. (2011). Postdialysis fatigue is associated with sedentary behavior. Clin. Nephrol. 75, 426–433 21543022

[B13] Hardy S. E. Studenski S. A. (2010). Qualities of fatigue and associated chronic conditions among older adults. J. Pain Symptom. Manage. 39, 1033–1042 10.1016/j.jpainsymman.2009.09.026 20538185 PMC2884149

[B14] Hedayati S. S. Yalamanchili V. Finkelstein F. O. (2012). A practical approach to the treatment of depression in patients with chronic kidney disease and end-stage renal disease. Kidney Int. 81, 247–255 10.1038/ki.2011.358 22012131 PMC3258342

[B15] Hettinga F. J. De Koning J. J. Schmidt L. J. Wind N. A. Macintosh B. R. Foster C. (2011). Optimal pacing strategy: from theoretical modelling to reality in 1500-m speed skating. Br. J. Sports Med. 45, 30–35 10.1136/bjsm.2009.064774 19850574

[B16] Jason L. A. Helgerson J. Torres-Harding S. R. Carrico A. W. Taylor R. R. (2003). Variability in diagnostic criteria for chronic fatigue syndrome may result in substantial differences in patterns of symptoms and disability. Eval. Health Prof. 26, 3–22 10.1177/0163278702250071 12629919

[B17] Jhamb M. Pike F. Ramer S. Argyropoulos C. Steel J. Dew M. A. Weisbord S. D. Weissfeld L. Unruh M. (2011). Impact of fatigue on outcomes in the hemodialysis (HEMO) study. Am. J. Nephrol. 33, 515–523 10.1159/000328004 21555875 PMC4484241

[B18] Johansen K. L. Doyle J. Sakkas G. K. Kent-Braun J. A. (2005). Neural and metabolic mechanisms of excessive muscle fatigue in maintenance hemodialysis patients. Am. J. Physiol. Regul. Integr. Comp. Physiol. 289, R805–R813 10.1152/ajpregu.00187.2005 15905222

[B19] Johansen K. L. Painter P. (2012). Exercise in individuals with CKD. Am. J. Kidney Dis. 59, 126–134 10.1053/j.ajkd.2011.10.008 22113127 PMC3242908

[B20] Johansen K. L. Painter P. L. Sakkas G. K. Gordon P. Doyle J. Shubert T. (2006). Effects of resistance exercise training and nandrolone decanoate on body composition and muscle function among patients who receive hemodialysis: a randomized, controlled trial. J. Am. Soc. Nephrol. 17, 2307–2314 10.1681/ASN.2006010034 16825332

[B21] Johansen K. L. Shubert T. Doyle J. Soher B. Sakkas G. K. Kent-Braun J. A. (2003). Muscle atrophy in patients receiving hemodialysis: effects on muscle strength, muscle quality, and physical function. Kidney Int. 63, 291–297 10.1046/j.1523-1755.2003.00704.x 12472795

[B22] Jolly M. (2005). How does quality of life of patients with systemic lupus erythematosus compare with that of other common chronic illnesses? J. Rheumatol. 32, 1706–1708 16142864

[B23] Kalantar-Zadeh K. Cano N. J. Budde K. Chazot C. Kovesdy C. P. Mak R. H. Mehrotra R. Raj D. S. Sehgal A. R. Stenvinkel P. Ikizler T. A. (2011). Diets and enteral supplements for improving outcomes in chronic kidney disease. Nat. Rev. Nephrol. 7, 369–384 10.1038/nrneph.2011.60 21629229 PMC3876473

[B24] Kallen A. J. Arduino M. J. Patel P. R. (2010). Preventing infections in patients undergoing hemodialysis. Expert Rev. Anti. Infect. Ther. 8, 643–655 10.1586/eri.10.47 20521893

[B25] Koufaki P. Mercer T. H. Naish P. F. (2002). Effects of exercise training on aerobic and functional capacity of end-stage renal disease patients. Clin. Physiol. Funct. Imaging 22, 115–124 10.1046/j.1365-2281.2002.00405.x 12005153

[B26] Kouidi E. Karagiannis V. Grekas D. Iakovides A. Kaprinis G. Tourkantonis A. Deligiannis A. (2010). Depression, heart rate variability, and exercise training in dialysis patients. Eur. J. Cardiovasc. Prev. Rehabil. 17, 160–167 10.1097/HJR.0b013e32833188c4 19745744

[B27] Kovacs A. Z. Molnar M. Z. Szeifert L. Ambrus C. Molnar-Varga M. Szentkiralyi A. Mucsi I. Novak M. (2011). Sleep disorders, depressive symptoms and health-related quality of life – a cross-sectional comparison between kidney transplant recipients and waitlisted patients on maintenance dialysis. Nephrol. Dial. Transplant. 26, 1058–1065 10.1093/ndt/gfq476 20685829

[B28] Krishnan A. V. Kiernan M. C. (2007). Uremic neuropathy: clinical features and new pathophysiological insights. Muscle Nerve 35, 273–290 10.1002/mus.20713 17195171

[B29] Letchmi S. Das S. Halim H. Zakariah F. A. Hassan H. Mat S. Packiavathy R. (2011). Fatigue experienced by patients receiving maintenance dialysis in hemodialysis units. Nurs. Health Sci. 13, 60–64 10.1111/j.1442-2018.2011.00579.x 21392194

[B30] Lim V. S. Flanigan M. J. (1989). The effect of interdialytic interval on protein metabolism: evidence suggesting dialysis-induced catabolism. Am. J. Kidney Dis. 14, 96–100 2757023 10.1016/s0272-6386(89)80183-0

[B31] Lindsay R. M. Heidenheim P. A. Nesrallah G. Garg A. X. Suri R. (2006). Minutes to recovery after a hemodialysis session: a simple health-related quality of life question that is reliable, valid, and sensitive to change. Clin. J. Am. Soc. Nephrol. 1, 952–959 10.2215/CJN.00040106 17699312

[B32] Marcora S. M. Staiano W. Manning V. (2009). Mental fatigue impairs physical performance in humans. J. Appl. Physiol. 106, 857–864 10.1152/japplphysiol.91324.2008 19131473

[B33] McCann K. Boore J. R. (2000). Fatigue in persons with renal failure who require maintenance haemodialysis. J. Adv. Nurs. 32, 1132–1142 10.1046/j.1365-2648.2000.01452.x 11114998

[B34] Micklewright D. Papadopoulou E. Swart J. Noakes T. (2010). Previous experience influences pacing during 20 km time trial cycling. Br. J. Sports Med. 44, 952–960 10.1136/bjsm.2009.057315 19364755

[B35] Noakes T. D. (2011a). Is it time to retire the A.V. Hill Model?: a rebuttal to the article by professor Roy Shephard. Sports Med. 41, 263–277 10.2165/11583950-000000000-00000 21425886

[B36] Noakes T. D. (2011b). Time to move beyond a brainless exercise physiology: the evidence for complex regulation of human exercise performance. Appl. Physiol. Nutr. Metab. 36, 23–35 10.1139/H10-082 21326375

[B37] Sakkas G. K. Ball D. Mercer T. H. Sargeant A. J. Tolfrey K. Naish P. F. (2003a). Atrophy of non-locomotor muscle in patients with end-stage renal failure. Nephrol. Dial. Transplant. 18, 2074–2081 10.1093/ndt/gfg237 13679483

[B38] Sakkas G. K. Sargeant A. J. Mercer T. H. Ball D. Koufaki P. Karatzaferi C. Naish P. F. (2003b). Changes in muscle morphology in dialysis patients after 6 months of aerobic exercise training. Nephrol. Dial. Transplant. 18, 1854–1861 10.1093/ndt/gfg237 12937235

[B39] Sakkas G. K. Gourgoulianis K. I. Karatzaferi C. Liakopoulos V. Maridaki M. D. Pastaka C. Lavdas E. Soher B. J. Dovas S. Fezoulidis I. Hadjigeorgiou G. M. Stefanidis I. (2008a). Haemodialysis patients with sleep apnoea syndrome experience increased central adiposity and altered muscular composition and functionality. Nephrol. Dial. Transplant. 23, 336–344 10.1093/ndt/gfn048 17890750

[B40] Sakkas G. K. Karatzaferi C. Zintzaras E. Giannaki C. D. Liakopoulos V. Lavdas E. Damani E. Liakos N. Fezoulidis I. Koutedakis Y. Stefanidis I. (2008b). Liver fat, visceral adiposity, and sleep disturbances contribute to the development of insulin resistance and glucose intolerance in nondiabetic dialysis patients. Am. J. Physiol. Regul. Integr. Comp. Physiol. 295, R1721–R1729 10.1152/ajpregu.00935.2007 18832089

[B41] Sarnak M. J. Levey A. S. Schoolwerth A. C. Coresh J. Culleton B. Hamm L. L. Mccullough P. A. Kasiske B. L. Kelepouris E. Klag M. J. Parfrey P. Pfeffer M. Raij L. Spinosa D. J. Wilson P. W. (2003). Kidney disease as a risk factor for development of cardiovascular disease. Hypertension 42, 1050–1065 10.1161/01.HYP.0000102971.85504.7c 14604997

[B42] Shephard R. J. (2009). Is it time to retire the “central governor”? Sports Med. 39, 709–721 10.2165/11315130-000000000-00000 19691362

[B43] Skein M. Duffield R. Edge J. Short M. J. Mundel T. (2011). Intermittent-sprint performance and muscle glycogen after 30 h of sleep deprivation. Med. Sci. Sports Exerc. 43, 1301–1311 10.1249/MSS.0b013e31820abc5a 21200339

[B44] Sklar A. Newman N. Scott R. Semenyuk L. Schultz J. Fiacco V. (1999). Identification of factors responsible for postdialysis fatigue. Am. J. Kidney Dis. 34, 464–470 10.1016/S0272-6386(99)70073-9 10469856

[B45] Stenvinkel P. (2010). Chronic kidney disease: a public health priority and harbinger of premature cardiovascular disease. J. Intern. Med. 268, 456–467 10.1111/j.1365-2796.2010.02269.x 20809922

[B46] Tryc A. B. Alwan G. Bokemeyer M. Goldbecker A. Hecker H. Haubitz M. Weissenborn K. (2011). Cerebral metabolic alterations and cognitive dysfunction in chronic kidney disease. Nephrol. Dial. Transplant. 26, 2635–2641 10.1093/ndt/gfq729 21216887

[B47] Ware J. E. Kristin K. Kosinski S. M. Gandek B. (1993). SF-36^®^ Health Survey Manual and Interpretation Guide. Boston, MA: Medical Center, The Health Institute

[B48] Wessely S. (2001). Chronic fatigue: symptom and syndrome. Ann. Intern. Med. 134, 838–843 11346319 10.7326/0003-4819-134-9_part_2-200105011-00007

[B49] Yatham L. N. Lecrubier Y. Fieve R. R. Davis K. H. Harris S. D. Krishnan A. A. (2004). Quality of life in patients with bipolar I depression: data from 920 patients. Bipolar Disord. 6, 379–385 10.1111/j.1399-5618.2004.00134.x 15383130

